# Expression of CD44v6 and Its Association with Prognosis in Epithelial Ovarian Carcinomas

**DOI:** 10.1155/2012/908206

**Published:** 2012-02-23

**Authors:** Dang-xia Zhou, Yun-xia Liu, Ya-hong Xue

**Affiliations:** ^1^Pathology Department, Medical School, Xi'an Jiaotong University, Xi'an 710061, China; ^2^Key Laboratory of Environment and Genes Related to Diseases, Ministry of Education, Xi'an 710061, China; ^3^Department of Obstetrics and Gynecology, Xi'an First Hospital, Xi'an 710002, China

## Abstract

The aim of this study was to evaluate CD44v6 protein expression and its prognostic value of CD44v6 in ovarian carcinoma. The expression of CD44v6 was analyzed in 62 patients with ovarian carcinoma by immunohistochemical method. The data obtained were analyzed by univariate and multivariate analyses. The present study clearly demonstrates that tumor tissues from 41 (66.1%) patients showed positive expression with CD44v6. The expression of CD44v6 was significantly correlated with histological type, FIGO stage and histological grade of ovarian carcinomas. Concerning the prognosis, the survival period of patients with CD44v6 positive was shorter than that of patients with CD44v6 negative (36.6% versus 66.7%, 5-year survival, *P* < 0.05). Univariate analysis showed that CD44v6 expression, histological type, FIGO stage and histological grade were associated with 5-year survival, and CD44v6 expression was associated with histological type, FIGO stage and histological grade and 5-year survival. In multivariate analysis, using the COX-regression model, CD44v6 expression was important prognostic factor. In conclusion, these results suggest that CD44v6 may be related to histological type, FIGO stage and histological grade of ovarian carcinomas, and CD44v6 may be an important molecular marker for poor prognosis in ovarian carcinomas.

## 1. Introduction

Ovarian carcinoma is one of the leading causes of cancer-related deaths from gynecological cancer and the seventh most common cancer worldwide [1]. Despite progress in detection and treatment, epithelial ovarian cancer is the most lethal gynecologic malignancy in women [2]. One important reason for high mortality caused by ovarian cancer is the poor understanding of the underlying biology, which in turn contributes to a lack of reliable biomarker for disease detection and effective therapeutic agents.

Adhesion processes are involved in all levels of the metastatic cascade. Most of the adhesion receptor families so far reported, including integrins, cadherins, selectins, immunoglobulins, and proteoglycans, have played a great role in various stages of tumor progression and metastasis [3].

CD44 family is one kind of important cell adhesion molecules. The CD44 gene is 50–60 kDa in size, resides on chromosome 11p13, and is known to be composed of at least 20 exons. CD44v6 is an important isoform of CD44 family [4–7]. It is a transmembrane glycoprotein widely distributed among different tissues and is a receptor of the extracellular matrix component hyaluronic acid [4]. Although the functions of CD44v6 in humans remain unclear, it plays an important role in the growth and metastasis development in several human tumors such as breast cancer [5, 6], colon cancer [7, 8], gastrointestinal caner [9, 10], lung cancer [11, 12], cervical cancer [13, 14], endometrial carcinoma [15], and bladder cancer [16]. CD44v6 was reported to associate with shorter survival of many tumors [17].

To date, however, the reports concerning the relationship between the expression of CD44v6 and prognosis of ovarian carcinoma are scare.

Therefore, the present study was designed to clarify possible role of adhesion molecule CD44v6 in ovarian carcinomas. In this study, we examined the immunohistochemical expression of CD44v6 in 62 cases of ovarian carcinoma and define the relationship between the expression of CD44v6 and clinicopathological parameters in ovarian carcinoma. Importantly, we investigated adhesion molecule CD44v6 expression for its prognostic value in ovarian carcinomas by univariate and multivariate analysis.

## 2. Materials and Methods

### 2.1. Patients

Women with a diagnosis of epithelial ovarian carcinoma were randomized and recruited to this cohort study. The exclusion criterion was inadequate follow-up data and chemotherapy or radiotherapy before operation. Finally, 62 patients were eligible and selected for the study. Formalin-fixed, paraffin-embedded blocks were retrieved from the pathologic archives of the Department of Pathology at the Affiliate Hospital of Xi'an Jiaotong University.

 The slides were evaluated by at least two pathologists. All discordant cases were reevaluated, and the result was defined by consensus. Tumors were surgically staged according to the International Federation of Gynecology and Obstetrics (FIGO) Staging Systems. Ovarian carcinomas were graded according to the histological degree of differentiated as well-differentiated (G1), moderately differentiated (G2), or as poorly differentiated (G3) carcinomas.

Of the 62 tumors, 45 were serous, 5 were mucinous, 8 were endometrioid, and 4 were undifferentiated. The clinicopathological parameters including age, menopausal status, nulliparous status, FIGO staging, and histological type and grade were evaluated.

### 2.2. Immunohistochemistry

Immunolocalization was performed using a streptavidin-biotin immunoperoxidase method according to the suppler's protocol (SP kit, Zhongshan, Beijing, China). Briefly, paraffin-embedded sections of formalin-fixed tissue sample were deparaffinized in xylene and rehydrated in graded concentrations of ethanol. After quenching the endogenous peroxidase acitivity with 0.3% H_2_O_2_, the sections were microwaved in 10 mmol/L sodium citrate (pH 6.0) for 15 min for antigen retrieval. After antigen retrieval, the 10% normal goat serum was applied to slides to eliminate nonspecific staining (endogenous background staining). The sections were then incubated with primary antibodies (Anti-CD44v6, Santa Cluz, CA, USA) for 90 min at 37°C, and after three successive rinsings with washing buffer (PBS), they were further incubated with biotinylated goat antimouse antibodies for 30 min at 37°C; after rinsing, the tissue sections were incubated with the horseradish peroxidase-  (HRP-) conjugated streptavidin for 20 min at room temperature. The slides were washed and stained with 3, 3′-diaminobenzidine (DAB) and then counter-stained with hematoxylin, dehydrated, and mounted with balsam for examination. Negative controls were obtained by omitting the primary antibody, substituted by PBS.

### 2.3. Interpretation of Immunohistochemistry

Positivity was scored semiquantitatively as CD44v6-negative (−), weakly (+), moderately (++), or strongly positive (+++). From no detectable expression to the faint expression in less than 5% of tumor cells was regarded as negative (−); CD44v6 expression between 5% and 25% of tumor cells was defined as weakly positive (+), CD44v6 expression between 25% and 50% of tumor cells was defined as moderately positive (++) and CD44v6 expression in over 75% of tumor cells was defined as strongly positive (+++).

For statistics, all the samples that expressed CD44v6 form (+) to (+++) were regarded as positive.

### 2.4. Statistical Analysis

All statistical comparisons were performed using SPSS version 13.0 software (SPSS Inc., Chicago, USA). For statistical analysis, the correlation between antigen expression and other clinicopathologic parameters was assessed by *χ* ^2^ test. Survival was calculated from the time of the primary operation. Survival rates were estimated by Kaplan-Meier statistics and Cox proportional hazards regression models, and survival curves were compared by using the Log-rank test. All the parameters were treated with univariate and multivariate analyses, and the *P* < 0.05 was regarded as statistically significant.

## 3. Results

### 3.1. Clinical Data

62 cases ovary carcinomas were successfully analyzed.  Table 1 summarizes the clinical and pathologic finding for all cases. The correlation between clinicopathologic features and 5-year survival rates showed that status of histological type, FIGO stages, and histological grades were significantly correlated with 5-year survival rate; see Table 1.

### 3.2. CD44v6 Expression

**Figure 1:**
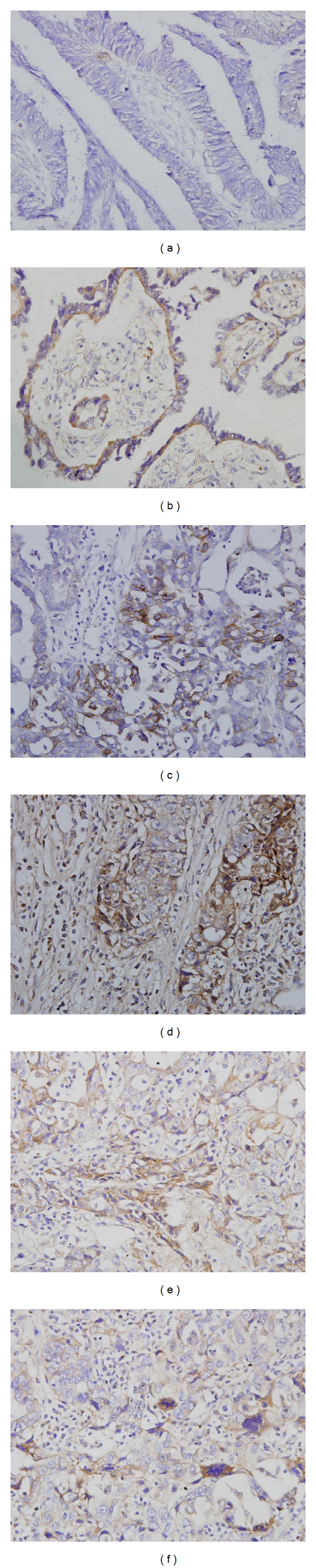
Immunohistochemical staining for CD44v6 protein in ovarian carcinomas, DAB ×400. (a) Negative control of ovarian carcinoma. (b–f) Membranous staining of tumor cells.

CD44v6 protein was preferentially expressed along the membrane of tumor cells. See Figure 1. Of 62 cases ovarian carcinomas, CD44v6 expression was recognized in 41 (66.1%) cases. Expression of CD44v6 was statistically correlated with histological grade, FIGO stage, and survival period of ovarian carcinomas, but not correlated with age, menopausal status, nulliparous status, and histological type. The correlation found between CD44v6 expression in ovarian carcinoma and clinicopathologic parameters is shown in Table 2.

### 3.3. Survival Analysis

**Figure 2:**
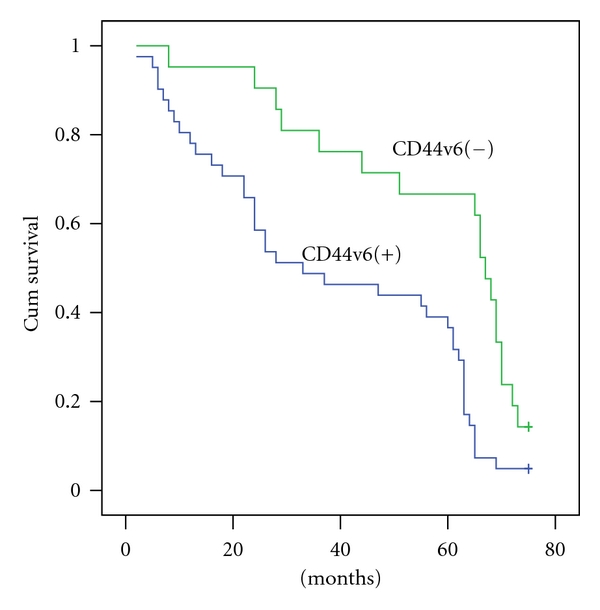
Overall survival curves of patients with CD44v6 positive (Blue line) and negative (Green line) ovarian carcinoma (*P* < 0.05).

The 5-year survival rate of the women in this study was 46.7% (29/62). A significance difference was found between CD44v6 expression and 5-year survival rate; see Table 2 (*P* < 0.05).  Figure 2 shows survival curves for the women with CD44v6-positive ovarian carcinoma and those with CD44v6-negative ovarian carcinoma. In the women with CD44v6-positive ovarian carcinoma and those with CD44v6-negative ovarian carcinoma, the 5-year overall survival rate was 51.7% and 78.8%, respectively. The prognosis of the women with CD44v6-positive ovarian carcinoma was poorer compared with that of women with CD44v6-negative ovarian carcinoma, and the women with CD44v6-negative ovarian carcinoma had a much longer survival period than those with CD44v6-negative ovarian carcinoma.

Our results suggested that CD44v6 might be a useful factor for evaluating the prognosis of the women with ovarian carcinoma. Cox proportional hazards regression models were used to evaluate the hazard ratio and indicated that FIGO stage, histological grade, nulliparous, and CD44v6 were significant among the seven prognostic factors (*P* < 0.05); see Table 3.

## 4. Discussion

CD44, a transmembrane glycoprotein, is the prominent cell surface receptor for hyaluronan [6]. CD44 has been implicated in cell-cell and cell-extracellular matrix attachment, lymphocyte homing hemopoiesis, cell motility, growth factors presentation, and matrix metalloproteinase association. CD44 gene is 50–60 kDa in size, resides on chromosome 11p13, and is known to be composed of at least 20 exons. Therefore, CD44 family exists in a number of isoforms, all products of a single gene and generated by alternative splicing of variant exons inserted into a single extracellular membrane-proximal site [1, 3]. They play an important role in cell growth and differentiation. Recently, CD44 has recently attracted considerable attention in tumor biology [4–17]. One of its variants, CD44v6 is involved in the production of experimental metastasis. Previous reports have indicated that the overexpression of CD44v6 was correlated with poor prognosis of human cancers [4, 16].

However, there have been few reports on the correlation between clinicopathological indices and CD44v6 expression in ovarian carcinoma [1]. On the basis of this background, we studied the correlation between the expression of CD44v6 and the prognosis of patients with ovarian carcinoma.

In the present study, CD44v6 was expressed in 66.1% of ovarian carcinomas. This result was similar to that of the previous study in other tumors such as breast cancer [6]. Our studies were the first to show that expression of CD44v6 was statistically correlated with histological grade, FIGO stage, and survival period of ovarian carcinomas, but not correlated with age, menopausal status, nulliparous status and histological type in West-north population of China. We found that the higher the grade of ovarian carcinomas, the higher the positive rate of CD44v6 expression.

Although there is a similarity in expression pattern between different ovarian carcinomas, there are some differences; a possible explanation for the difference may be that ovarian carcinomas are heterogeneous entities, some derived from borderline tumors and others arising de novo.

Concerning prognosis, we found that the expression of the CD44v6 was significantly associated with increased mortality. The patient with CD44v6 positive tumors had a shorter survival than those with CD44 negative tumors. The 5-year survival rate of CD44v6 positive was 36.6% but that of negative was 66.7%. These results suggested that downregulation of CD44v6 was associated with a good prognosis in patient with ovarian carcinoma. These indicated that CD44v6 might be an important and useful marker for indicating a poor prognosis in women with ovarian carcinoma.

Differing methods were used in previous studies in which the relationship between CD44v6 expression and prognosis was investigated. For example, Yamada et al. [18] quantitatively analyze the expression of CD44v6 in colon cancer using a real-time reverse transcriptase-polymerase chain reaction (RT-PCR) approach. They also found that CD44v6 expression was an important prognostic factor for colon carcinoma.

In conclusion, CD44v6 is considered to be correlated with histological grade, FIGO stage, and survival period of ovarian carcinomas and to play an important role in predicating the prognosis of patients with ovarian carcinomas. CD44v6 may become an important prognostic factor in ovarian carcinoma. However, further studies are needed to fully elucidate the regulatory mechanisms of CD44 variant expression, and further studies using a large sample and longer follow-up are necessary to verify these results.

## Figures and Tables

**Table 1:** Correlation of the clinicopathologic features and 5-year survival rates.

Clinicopathologic parameters	Number of cases	5-years survival cases (%)	*P* value
Age *⩽* 50 > 50	21 41	10 (47.6) 19 (46.3)	> 0.05

Menopausal status Premenopausal Postmenopausal	17 45	7 (41.2) 22 (48.9)	> 0.05

Nulliparous status Yes No Serous	9 53 45	3 (33.3) 26 (49.1) 26 (57.8)	> 0.05

Histological type Mucinous Endometrioid Undifferentiated	5 8 4	1 (20) 2 (25) 0 (0)	< 0.05

FIGO stage I II III IV	16 23 15 8	14 (87.5) 9 (39.1) 4 (26.7) 2 (25)	< 0.05

Grade I II III	19 32 11	15 (78.9) 11 (34.4) 3 (27.3)	< 0.05

**Table 2:** Correlation between CD44v6 protein expression and various clinicopathologic parameters.

Clinicopathologic parameters	Number of cases	CD44v6-positive cases (%)	*P* value
Age *⩽* 50 > 50	21 41	13 (61.9) 28 (68.3)	> 0.05

Menopausal status Premenopausal Postmenopausal	17 45	11 (64.7) 30 (66.7)	> 0.05

Nulliparous status Yes No Serous	9 53 45	6 (66.7) 35 (66.0) 32 (71.1)	> 0.05

Histological type Mucinous Endometrioid Undifferentiated	5 8 4	2 (40) 3 (37.5) 4 (100)	> 0.05

FIGO stage I II III IV	16 23 15 8	5 (31.2) 18 (78.2) 11 (73.3) 7 (87.5)	< 0.05

Grade I II III	19 32 11	6 (52.6) 25 (65.6) 10 (90.9)	< 0.05

Survival period ≤ 5 > 5	33 29	26 (78.8) 15 (51.7)	< 0.05

**Table 3:** Multivariate analyses of prognostic for survival of women with ovarian carcinomas.

Factors	Regression coefficient	Relative risk	*P*
Age	0.778	2.177	> 0.05
Menopause status	0.504	1.655	> 0.05
Nulliparous	1.207	3.343	< 0.05
Histological type	0.375	1.455	> 0.05
FIGO stage	0.465	1.592	< 0.05
Grade	0.685	1.984	< 0.05
CD44v6	1.926	6.849	< 0.05
